# Age of puppies at referral to veterinary cardiology specialists for murmur investigation

**DOI:** 10.1186/s13028-021-00603-0

**Published:** 2021-09-23

**Authors:** Lynn Bernadette Rovroy, Viktor Szatmári

**Affiliations:** Department of Clinical Sciences, Faculty of Veterinary Medicine, Utrecht University, Yalelaan 108, 3584 CM Utrecht, the Netherlands

**Keywords:** Auscultation, Cardiac, Congenital, Heart, Screening

## Abstract

**Background:**

Cardiac auscultation is an important screening test at the first health examination of puppies because most clinically relevant congenital cardiac anomalies cause a loud murmur from birth. This retrospective study aimed to investigate the age at which dogs with suspected congenital cardiac anomalies were referred to a veterinary cardiology specialist for murmur investigation. A secondary aim was to establish the time interval between the visit to the cardiologist and the first available murmur documentation. The digital archive of a veterinary teaching hospital was searched for dogs with congenital cardiac anomalies and puppies with innocent murmurs during a 5-year period. Dogs had to be referred because of a murmur, and they had to undergo physical examination and echocardiography by a veterinary cardiology specialist. The health certificate section of the pet passport, and the medical records from the referring veterinarian, were reviewed to identify the date when the murmur was first documented.

**Results:**

Of the 271 included dogs, 94% had a congenital cardiac anomaly and 6% had an innocent murmur. The dogs’ median age was 190 days when they were examined by the cardiologist. Only 10% of the dogs were referred by the breeder’s veterinarian, while 90% of the dogs were referred by the new owner’s veterinarian. The median age of the first available murmur documentation by a first opinion veterinary practitioner was 95 days.

**Conclusions:**

Only 10% of the puppies in the present study were referred to a veterinary cardiology specialist for murmur investigation before they were sold to a new owner. Referral prior to re-homing would have been feasible if the murmur had been detected and documented by the breeder’s veterinarian, if referral was offered by the breeder’s veterinarian and the referral was accepted by the breeder.

## Background

Most puppies with a congenital heart disease show no clinical signs of their heart disease when they are examined by a first opinion veterinary practitioner at the first veterinary health screening at about 6–8 weeks of age [1–11]. The presence of a cardiac murmur at the screening examination is often the only clue that a congenital cardiac anomaly might be present [1–11]. Although the prevalence of congenital cardiac anomalies in the general mixed breed canine population is very low [10], non-pathological cardiac murmurs are common findings in clinically healthy puppies at the age of about seven weeks (15–31%) [11–13]. Differentiating pathological from non-pathological murmurs can be challenging, if not impossible, based on auscultation alone, especially in cases of soft systolic murmurs [1, 11]. This initial differentiation is, however, crucial in deciding whether or not to recommend referral of a puppy with a suspected pathological murmur to a veterinary cardiology specialist, since congenital cardiac anomalies can be associated with high morbidity and mortality [2–5, 7–9, 14–16]. Ideally, the cause of a cardiac murmur is established before the breeder sells the puppy to a new owner at about 8–9 weeks of age [3].

Of the three most prevalent congenital cardiac anomalies in dogs (i.e., aortic stenosis, patent ductus arteriosus and pulmonic stenosis), both left-to-right shunting patent ductus arteriosus and severe pulmonic stenosis are effectively treatable conditions and they typically cause loud murmurs [2, 7, 9, 14–18]. The younger a dog is when the occlusion of a left-to-right shunting patent ductus arteriosus takes place, the greater the chance that the defect can be corrected, and with this the long term cardiac morbidity and mortality can be substantially reduced [2, 9, 14, 16]. Once cardiac related symptoms appear (resulting from congestive left-sided heart failure or pulmonary hypertension), the prognosis becomes much worse [9, 14, 17]. The same holds true for severe congenital valvular pulmonic stenosis, where asymptomatic dogs have a better prognosis after a successful balloon valvuloplasty compared to dogs that already show cardiac-related clinical signs [7, 15, 18]. In addition, balloon valvuloplasty becomes technically more challenging and more expensive in full-grown large-breed dogs because a large-diameter balloon or a double-balloon technique might be required for the effective dilatation of the large diameter vessels [19]. Also, with advancing secondary right ventricular concentric hypertrophy, there are more potential perioperative complications, such as post ballooning right ventricular outflow tract obstruction (so called “suicide right ventricle”), and a suboptimal long term outcome might follow because of irreversible right ventricular remodelling. Last but not least, selling a puppy with an unrevealed congenital cardiac anomaly can have remarkable emotional and financial consequences for the new owner and for the breeder, as well as legal sequelae for the breeder’s veterinarian who performed the first health screening [3].

Several studies report diagnostic and therapeutic findings in adult dogs with congenital cardiac anomalies [17, 20, 21]. However, no studies focus on the age when dogs with a suspected congenital cardiac anomaly are referred to a veterinary cardiology specialist for murmur investigation and subsequent therapy. In addition, no studies have investigated the time interval (i.e., amount of delay) between the referral to a cardiologist and the first available murmur documentation by a first opinion veterinary practitioner. This retrospective study aimed to investigate these questions.

## Methods

In this retrospective case series, the electronic database of a single academic veterinary teaching hospital was searched for puppies with an innocent murmur or all dogs with congenital cardiac anomalies, during a period of five years, between January 2014 and January 2019. Inclusion criteria were: (1) dogs had to be referred, either solely for a cardiac murmur or a cardiac murmur in combination with other problems, to the cardiology service of the authors’ institution; and (2) a physical examination followed by an echocardiographic examination had to be performed by an EBVS^®^ (European Board of Veterinary Specialization) recognized European veterinary specialist in small animal cardiology. An upper age limit of 10 months was applied in dogs with a non-pathological (innocent) murmur for inclusion [13]. No age limitation was applied in dogs with congenital cardiac anomalies. Exclusion criteria were: (1) dogs where the veterinary cardiology specialist diagnosed a congenital cardiac anomaly, but the dog was brought to the clinic for screening, without a referral by another veterinarian; and (2) dogs where the veterinary cardiology specialist diagnosed a congenital cardiac anomaly, but the reason for referral was not, or did not include, a previously recognized cardiac murmur.

All dogs underwent a thorough physical examination and an echocardiogram both performed by a veterinary cardiology specialist. The echocardiogram was carried out on unsedated animals restrained in right and left lateral recumbency, and the examination consisted of 2-dimensional, M-mode, color Doppler and spectral (pulsed- and continuous wave) Doppler studies from the standard left and right parasternal as well as subcostal views [22].

An attempt was made to obtain the following information: (1) the age of the dog at the first documentation of the murmur in the health certificate section of the dog’s pet passport, (2) the age of the dog at the first documentation of the murmur in the medical record and/or in the referring letter of the referring veterinarian, (3) whether the dog was referred by the breeder’s or the new owner’s veterinarian, (4) the age of the dog at the visit to a non-cardiologist veterinarian for an echocardiographic examination (if this took place), (5) the age of the dog at the visit to the cardiology service of the authors’ institution, (6) the intensity of the murmur as described by the referring veterinarian, (7) the intensity of the murmur as described by the attending veterinary cardiology specialist, (8) the echocardiographic diagnosis made by the non-cardiologist, (9) the echocardiographic diagnosis made by the veterinary cardiology specialist, and (10) the presence or absence of clinical signs, as well as the leading clinical sign in case of symptomatic dogs as described by the owner. If a dog showed more than one clinical sign, it was classified only in a single category based on the following priority order: distended abdomen, dyspnea, syncope, cough, retarded growth, exercise intolerance and weight loss. For example, if a dog had dyspnea and exercise intolerance, the case was listed under dyspnea.

The murmur intensity as recorded by the attending veterinary cardiology specialist on the day of the visit was compared with the historical recorded findings of the referring veterinarian. The auscultation findings of the referring veterinarian were identified in the medical records which were sent to the authors’ institution as part of the referring letter. The referring veterinarian was either the breeder’s veterinarian or the new owner’s veterinarian. The findings of the breeders’ veterinarians were obtained from the health certificate section in the pet passport, if this document was available, in case the puppy was referred by the new owner’s veterinarian. Pet passports in the country where the study took place contain a separate section about the health status of each organ, including the heart, which should be filled in by a first opinion veterinary practitioner at each time when a vaccination is given or before an animal travels abroad. Though completing this document is not obligatory, it is part of good veterinary practice. Moreover, this is the only written veterinary documentation that serves as a health certificate when a puppy is sold to the new owner.

Based on the veterinary cardiology specialist’s echocardiographic diagnosis, the murmurs were classified as either pathologic or non-pathologic. Besides identifying the cause of the murmurs, the need and possibility for interventional/surgical correction of the defect were noted. The severity of obstructive anomalies (such as pulmonic stenosis, aortic stenosis and double-chambered right ventricle) was classified based on the Doppler-derived pressure gradient in unsedated, awake animals as severe (higher than 80 mmHg), moderate (between 50 and 80 mmHg), or mild (between 16 and 49 mmHg) [15, 18, 23].

The veterinary cardiology specialist’s final diagnosis was compared to the non-cardiologist’s echocardiographic diagnosis, if the dog had undergone an echocardiographic examination elsewhere before referral to the authors’ institution. The findings of the two veterinarians were classified as full agreement, partial agreement or disagreement based on therapeutic consequences. An example of full agreement is when both assessors diagnosed the same disorder with the same category of severity, even if the pressure gradient of an obstructive disorder differed markedly (e.g., pulmonic stenosis with a pressure gradient of 90 mmHg versus 190 mmHg), but the difference did not affect the therapeutic advice, because in both cases balloon valvuloplasty would be advised. An example of a partial agreement is when the same defect was found by both examiners but the severity was different (e.g., moderate versus severe pulmonic stenosis), even if the absolute difference was smaller than in the previous example. When the veterinary cardiology specialist found multiple congenital cardiac anomalies and the non-cardiologist detected only some of them, it was also marked as a partial agreement. However, if the natural history of the disease was assumed to lead to a different diagnosis (i.e., first left-to-right shunting patent ductus arteriosus and later a right-to-left shunting one) between the two examinations, then the case was marked as a full agreement. The degree of agreement between the different assessors was expressed in percentages.

Descriptive statistical analysis was performed using commercial statistical software (R studio 0.98.1073, Boston, MA and Microsoft Office Excel 2018, Microsoft Corp, Redmond, WA). The age of the dogs is presented in days as median and range. Correlation between murmur intensity and the age at referral was calculated using Pearson’s correlation coefficient. A correlation coefficient close to  +  1 or − 1 indicated positive or negative correlation, respectively, whereas a value close to 0 indicated no correlation.

## Results

### Animals

**Fig. 1:**
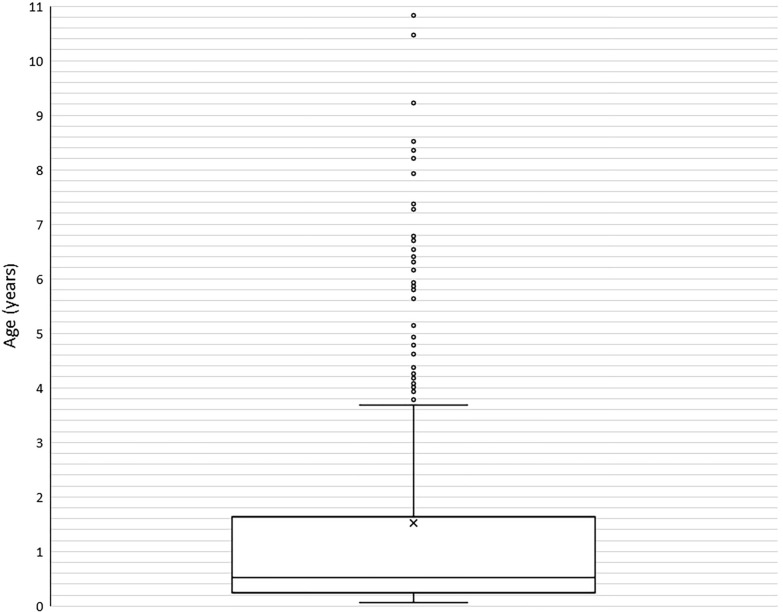
This box whisker plot shows the age of the 271 dogs in days on the vertical axis when they were examined by a veterinary cardiology specialist because of a cardiac murmur. The box represents the interquartile range and the line within the box represents the median age of the dogs at referral: 190 days (6.3 months). The x represents their mean age (1.5 years). Dogs with the youngest and oldest age at referral are shown with the whiskers and the outliers are displayed with circles

A total of 271 dogs fulfilled the inclusion criteria. The median age of the dogs when presented to the cardiology service of the authors’ institution for investigation of a cardiac murmur was 190 days (6.3 months, range 25 days–10 years and 10 months) (Fig. 1).

Of the 271 dogs 254 (94%) had a pathological murmur and 17 (6%) had a non-pathological (innocent) murmur. According to the owners, 182 dogs (67%) were clinically healthy, while 89 (33%) showed one or more clinical signs: exercise intolerance (n  =  28), syncope (n  =  18), dyspnea (n  =  16), cough (n  =  16), distended abdomen (n  =  7), retarded growth (n  =  3), and weight loss (n  =  1).

Only three dogs with cardiac-related clinical signs resulting from severe congenital cardiac anomalies that were not referred solely or in part because of a murmur were identified in the study period. These dogs were excluded from the study population, as the murmur was detected at the authors’ clinic for the first time. One of these three dogs had severe tricuspid valve dysplasia, the second dog had severe mitral valve dysplasia and the third one had both a severe mitral and a severe tricuspid valve dysplasia.

### Murmur documentation by first opinion veterinary practitioners

The 271 dogs were referred from 224 different veterinary practices. Written documentation about the presence of a murmur from a first opinion veterinary practitioner was available in 249 of the 271 dogs (92%). These 249 dogs’ median age was 95 days (3.2 months) at the first available murmur documentation. The source of this information was either the pet passport (when this was available) or the medical record of the referring veterinarian, recorded either by the breeder’s or new owner’s veterinarian. Inconsistences regarding the documented dates in the two types of documents are shown in Table 1.

**Table 1:** Inconsistent documentation of auscultation findings in 271 dogs with a cardiac murmur in two medical documents issued by the referring veterinarians: the medical record and the health certificate section of the pet passport

Included dogs								
n = 271								
Only medical record was available		Both medical record and pet passport were available				Only pet passport was available		Both medical record and pet passport were unavailable
n = 185		n = 78				n = 1		n = 7
Murmur was recorded	Murmur was not recorded	Murmur was recorded only in pet passport	Murmur was recorded only in medical record	Murmur was recorded in medical record and pet passport	Murmur was recorded neither in medical record nor in pet passport	Murmur was recorded in pet passport	Murmur was not recorded in pet passport	–
n = 173	n = 12	n = 1	n = 26	n = 49	n = 2	n = 0	n = 1	

Based on the referring veterinarians’ medical records only (n  =  264), the median age of the dogs when their murmur was first documented was 99 days (3.3 months, range 4 days–10 years and 10 months). The delay between the first available murmur documentation and the visit to the cardiology specialist was 95 days (3.2 months, range 0 day–6 years and 9 months).

**Fig. 2:**
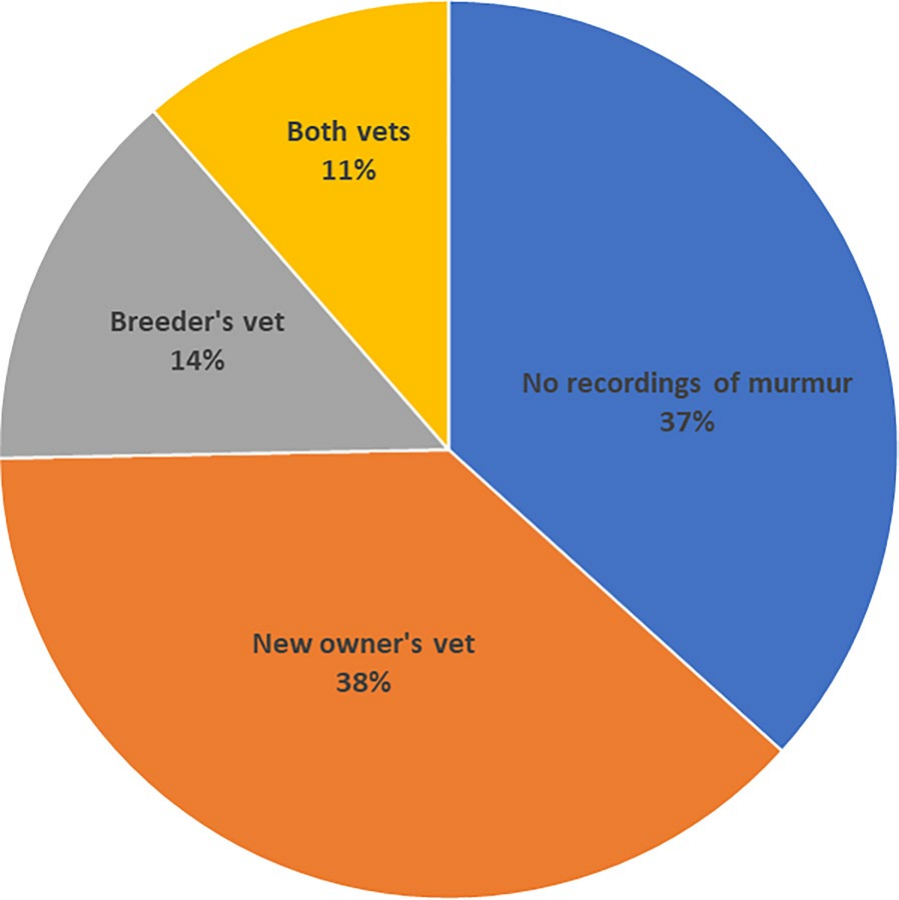
This graph shows whether the breeder’s and/or the new owner’s veterinarian documented the presence of a cardiac murmur in the health certificate page of the 79 available pet passports. In only 25% of the cases was the presence of a murmur documented by the breeder’s veterinarian

In only 50 of the 79 available pet passports (63%) was the presence of the cardiac murmur recorded. All passports were of Dutch origin. The median age when the murmur was first documented in these 50 dogs was 67 days (2.2 months, range 41 days–7 years and 3 months). Whether the breeder’s or the new owner’s veterinarian documented the murmur is shown in Fig. 2. The median age of those 20 dogs, where the breeder’s veterinarian recorded the murmur in the pet passports was 45 days (1.5 month, range 41–87 days). In 66 of the 79 available passports (84%) the date of the first vaccination was available. The median age of the dogs at the first vaccination was 45 days of age (1.5 month, i.e., 6.4 weeks, range 36–423 days).

Only 27 of the 271 dogs (10%) were investigated for their murmur at the cardiology service of the authors’ institution based on the referral from the breeder’s veterinarian. Of these dogs, the median age at the first available documentation of the murmur was 46 days (range 25–73 days) and the median age at referral was 52 days (range 25–109 days).

### Cause of murmur

The veterinary cardiology specialists diagnosed isolated congenital cardiac anomalies in 211 dogs (83% of all dogs with a pathologic murmur) as the cause of the murmur (Table 2). Multiple congenital cardiac anomalies were found in 43 dogs (17% of all dogs with a pathologic murmur): 34 dogs (79%) were diagnosed with two cardiac anomalies, 7 dogs (16%) with three ones, and 2 dogs (5%) with four anomalies.

**Table 2:** Frequency of isolated cardiac anomalies found by the veterinary cardiology specialist as the cause of murmur

Congenital anomaly	Number of dogs
Aortic stenosis	64
Severe	17
Moderate	8
Mild	39
Pulmonic stenosis	59
Severe	41
Moderate	15
Mild	6
Patent ductus arteriosus	44
Left-to-right shunting	41
Right-to-left shunting	3
Mitral valve dysplasia	18
Ventricular septal defect	13
All left-to-right shunting	
Tricuspid valve dysplasia	8
Double chambered right ventricle	5
All moderate	

The cause of murmur in the 27 dogs that were referred by the breeder’s veterinarian is shown in Table 3.

**Table 3:** Cause of murmur in 27 dogs referred by the breeder’s veterinarian to the veterinary cardiology specialist

Cause of murmur	Number of dogs
Patent ductus arteriosus	7
All left-to-right shunting	
Pulmonic stenosis	6
Severe	3
Moderate	2
Mild	1
Ventricular septal defect	4
All left-to-right shunting	
Aortic stenosis	2
Both moderate	
Innocent murmur	2
Double chambered right ventricle	1
Mitral valve dysplasia	1
Tetralogy of Fallot	1
Pulmonic stenosis	
Severe, in combination with	
Ventricular septal defect	
Right-to-left shunting	1
Moderate, in combination with	
Mitral valve dysplasia	1
Patent ductus arteriosus	
Left-to-right shunting, in combination with	
Pulmonic stenosis	
Moderate	1

### Agreement in the presence and intensity of the murmur

Three veterinary cardiology specialists were involved in the assessment of the dogs at the authors’ institution. The physical examination and the echocardiograms on the same dog were carried out by the same veterinary cardiology specialist on the same day. The veterinary cardiology specialist heard no murmur in only two of the 271 referred dogs. One of these two dogs was diagnosed with a right-to-left shunting patent ductus arteriosus on the day of referral and previously with a left-to-right shunting one. The other dog had most likely a non-pathological murmur, which resolved spontaneously, as echocardiography revealed no abnormalities at all on the day of referral. Another explanation to the disappearance of the murmur could be transient dynamic left ventricular outflow tract obstruction caused by systolic anterior motion of the mitral valve.

**Fig. 3:**
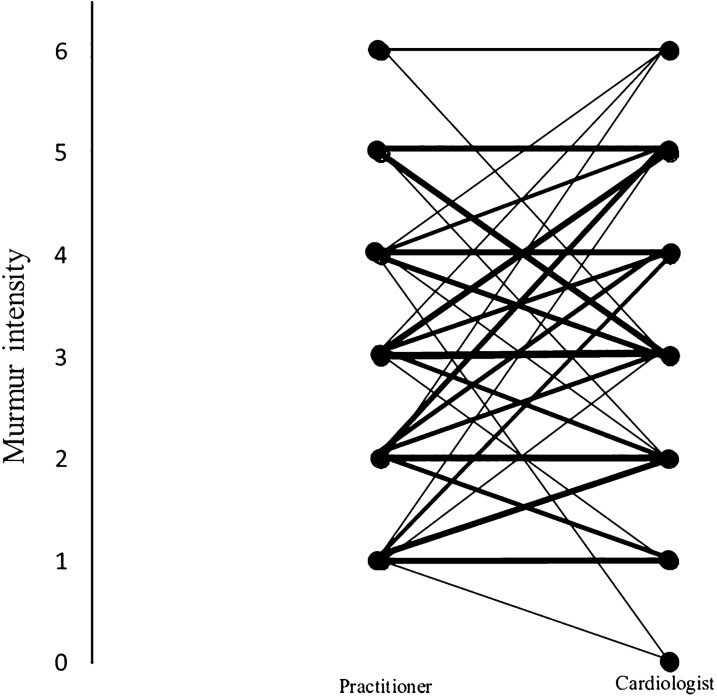
Comparison of murmur intensity in 139 dogs, in which the cardiac murmur was quantified on a 1–6 scale both by the referring veterinarian (Practitioner) and by the veterinary cardiology specialist (Cardiologist) at two different time points. The thickness of the lines is proportional to the number of cases. In 39 (28%) cases there was no difference between their judgements, in 53 (38%) cases one grade of difference, in 28 (20%) cases two grades of difference, in 15 (11%) cases three grades of difference and in 4 (3%) cases four grades difference were found. The mean murmur intensity as recorded by the referring veterinarian was 2.7 (range 1–6), while this value was 3.3 (range 0–6) as assessed by the veterinary cardiology specialists. 0  =  absence of an audible murmur, 1  =  softest murmur, 6  =  loudest murmur intensity

**Fig. 4:**
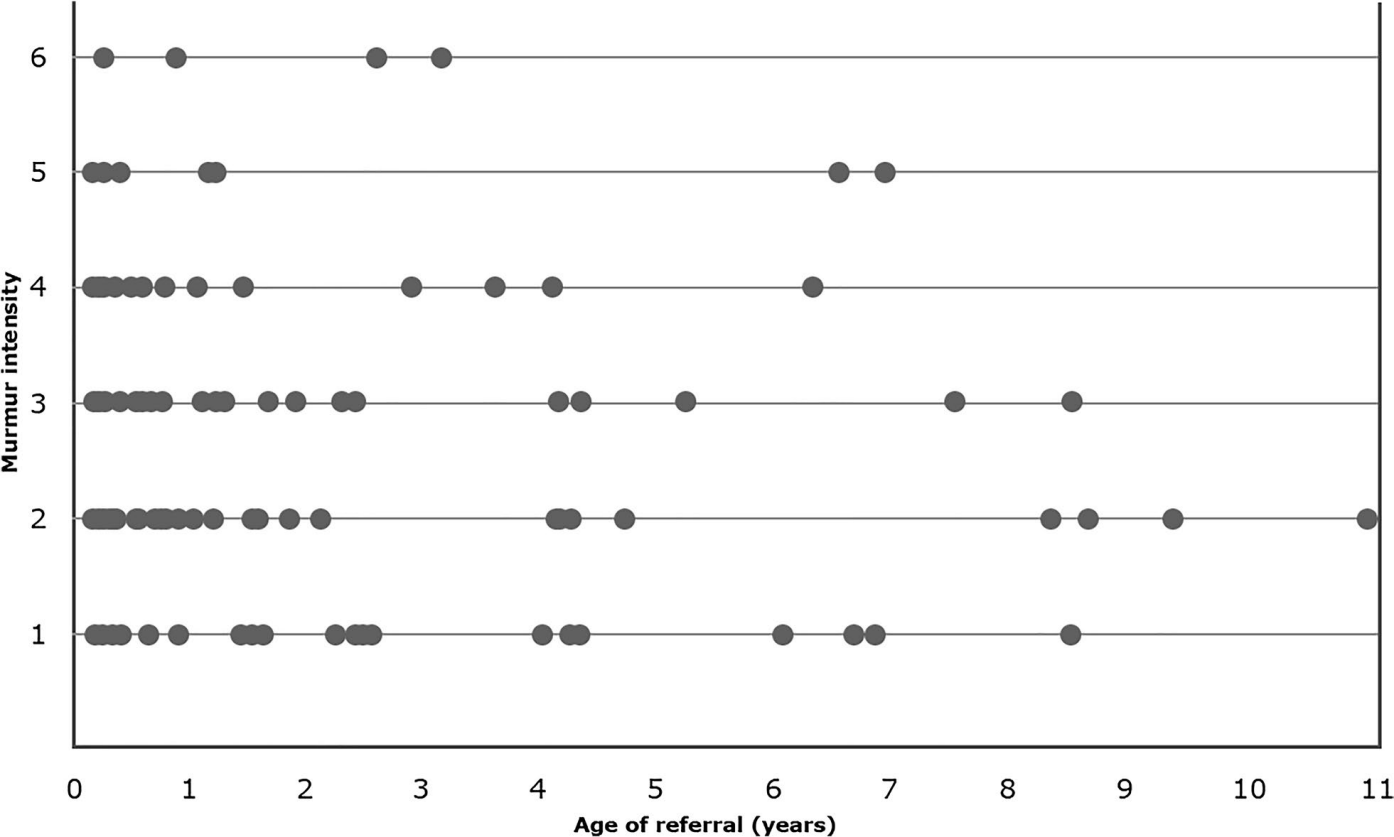
Comparison of the intensity of the cardiac murmur (on a 1–6 scale) as recorded by the referring first opinion veterinary practitioner and the age of 139 dogs (in years) when they were referred to the veterinary cardiology specialist for murmur evaluation. No correlation was found between the murmur intensity and the age at referral (correlation coefficient  =  -0.1). 0  =  absence of an audible murmur, 1  =  softest murmur, 6  =  loudest murmur

In the medical records of the referring veterinarians, the murmur was quantified on a 1–6 scale by 139 of the 248 veterinarians (56%) (Fig. 3), while the remaining 109 veterinarians either did not quantify the murmur intensity (n  =  66, 27%) or used a semi-quantitative assessment (i.e., loud or soft, n  =  43, 17%). The intensity of the murmur as assessed by the referring veterinarian did not show a correlation with the age at referral (correlation coefficient of − 0.10) (Fig. 4).

### Agreement in the echocardiographic diagnosis

An echocardiogram was performed by a non-cardiologist in 124 of the 271 dogs (46%) before the dog was referred to the authors’ institution. An agreement on the diagnosis was found in 65 of the 124 dogs (52%), a partial agreement in 15 (12%) and 44 (35%) dogs the non-cardiologist did not manage to reach a diagnosis or there was a disagreement. The median age of the dogs when the echocardiogram was performed by the non-cardiologist was 152 days (5.1 months, range 28 days–8 years and 4 months) and the median age when the echocardiogram was performed by the veterinary cardiology specialist was 190 days (6.3 months, range 25 days–10 years and 10 months). The median difference between the two echocardiograms was 38 days (5.4 weeks, range 1 day–5 years and 5 months).

### Treatable cardiac anomalies

In 95 of the 271 dogs (35%) the veterinary cardiology specialist recommended an intervention either to close a left-to-right shunting patent ductus arteriosus or to dilate a severe (or in selected cases a moderate) pulmonic stenosis. In 62 of the 95 dogs (65%), the owners agreed to perform cardiac intervention, which is 23% of all enrolled dogs. All procedures were performed at the authors’ institution.

The age of referral in these 95 dogs showed no correlation with the murmur intensity either. The correlation coefficient was − 0.12 when the murmur intensities of the cardiologists were used, and it was − 0.27 when the murmur intensities of the referring veterinarians were used for the analysis.

A left-to-right shunting patent ductus arteriosus was closed in 27 dogs, with trans-catheter embolization being used in 25 dogs and via thoracotomy in 2 dogs. The median age of these 27 dogs at the time of surgery was 115 days (3.8 months, range 68 days–8 years and 6 months), and the median age when their defect was diagnosed at the authors’ institution was 104 days (3.5 months, range 51 days–8 years and 6 months). The time interval between the diagnosis and therapy for an average dog was 11 days.

In 35 dogs a balloon valvuloplasty of a severe (or moderate) pulmonic stenosis was performed. The median age of these 35 dogs when they underwent a balloon valvuloplasty was 199 days (6.6 months, range 82 days–6 years and 7 months), and the median age when their pulmonic stenosis was diagnosed at the authors’ institution was 145 days (4.8 months, range 47 days–6 years and 6 months). The time interval between the diagnosis and balloon valvuloplasty for an average dog with pulmonic stenosis was 54 days.

## Discussion

One of the most important findings of the present study is that only 10% of the 271 dogs, in which the referring veterinarian suspected a congenital cardiac anomaly based on the presence of a cardiac murmur, were referred to a veterinary cardiology specialist before the dog was sold to a new owner. The remaining dogs were referred by the new owner’s veterinarian. Whether the breeders had informed the new owners about the presence of a cardiac murmur was not investigated. Because the cardiac murmur of most clinically relevant congenital cardiac anomalies are audible from birth, diagnosing congenital cardiac anomalies should be possible when the puppy is still in the breeder’s possession [1–5]. This turned out to be possible in several puppies, whose murmur was documented at the same time when the first vaccination was given (at a median age of 6.4 weeks) and their heart disease was subsequently diagnosed by a cardiology specialist at the median age of 7.4 weeks. The second vaccination is typically given at 9 weeks of age, when the pup is in the possession of the new owner. This means that if a murmur is missed at the first veterinary health screening at 6 weeks of age, or in case it was detected, but the breeder failed to see a veterinary cardiologist before the puppy is sold to a new owner, then the new owner will face all the emotional and financial consequences of a new puppy with a potential life-threatening health issue.

In the country where the study took place, the health certificate section in the pet passport is the only veterinary documentation available for new owners when they buy a puppy from a breeder. Pathological murmurs caused by congenital cardiac anomalies should have been present in every dog at the first health screening, which should be routinely carried out by the breeder’s veterinarian at 6 weeks of age, coinciding with the first vaccination. In the present case series, however, only 25% of the available health certificates of the pet passports contained a note about the presence of a murmur recorded by the breeder’s veterinarian. Possible reasons for this finding remain speculative and include the following options: (1) the breeder’s veterinarian did not notice the murmur, (2) the health certificate sections of the pet passports were filled in without having examined the puppy, (3) the breeder’s veterinarian did hear the murmur, but (intentionally or unintentionally) failed to record it in the pet passports.

Though the presence of a murmur was recorded in the medical records of the referring veterinarian in 94% of the cases, the pet passports contained this information in only 63% of the cases. This finding suggests that the medical records seem to be a more reliable source of information than the pet passports. However, medical records are not accessible to the pet owners who are about to buy a puppy from a breeder. The reason for the inconsistent documentation of murmurs by first opinion practicing veterinarians remains unclear.

Because louder murmurs are more likely to be pathologic, a younger age at referral would be expected in dogs with a loud murmur. However, the intensity of the murmur as judged by the referring veterinarian did not show a correlation with the age at referral. The two most common anomalies for which effective surgical correction is also available, severe pulmonic stenosis and hemodynamically relevant left-to-right shunting patent ductus arteriosus, are typically associated with a loud murmur. Even in this subgroup of dogs no correlation was found between the age at referral and the murmur intensity. Whether an owner eventually makes an appointment with a veterinary cardiology specialist or not, and if so when, depends not only on the referring veterinarian’s advice, but also the owner’s perceptions, motivation and financial background. This aspect was not investigated in the present study. However, the authors believe that it is important that a first opinion veterinary practitioner carefully performs cardiac auscultation and in case of a murmur recommends immediate referral to a veterinary cardiology specialist. This is the only way to prevent legal issues for the breeder and make sure that a reliable diagnosis is reached together with a prognosis and a sound therapy advice.

A non-pathologic murmur was found in only 6% of the referred dogs. The reason for this low number could be that the median age of the population at referral was higher (6.3 months) than the age when most innocent murmurs spontaneously resolve in puppies (median age of 3 months) [13]. In addition, the intensity of innocent murmurs is low, which makes their recognition more challenging [12]. It is also possible that first opinion veterinary practitioners did not recommend referral of puppies with soft systolic murmurs because they assumed that the murmur was most likely innocent based on the auscultation, or the puppies were first referred to a non-cardiologist for an echocardiogram where no abnormalities were found.

In the 5-year period of the present study, only three dogs were identified whose murmur was caused by a congenital heart defect and not recognized before the referral. This finding suggests that the first opinion veterinary practitioners in the Netherlands are good at recognizing cardiac murmurs, at least in cases when they examined individual animals. However, when health checks of litters were performed by the breeders’ veterinarians at the first health screening, the number of documented cardiac murmurs was low. This can be explained by the fact that screening an individual puppy is easier than a whole litter, especially if the litter screening takes place at the breeder’s home, under suboptimal circumstances instead in a quiet consultation room.

Congenital cardiac anomalies can lead to morbidity and mortality [2–5, 7–9, 14–19]. Of the three most prevalent congenital cardiac anomalies in dogs, both left-to-right shunting patent ductus arteriosus and severe pulmonic stenosis are effectively treatable conditions [2, 7, 9, 14–19, 23–33]. The younger a dog with a congenital cardiac anomaly, the greater the chance that the defect can be corrected without negative effect on the longevity and the quality of life [2, 9, 14, 16]. In severe congenital valvular pulmonic stenosis, dogs that show no clinical signs have a better prognosis after balloon valvuloplasty compared to dogs that already showed cardiac-related signs before intervention [15, 18]. In the present study, one third of the dogs already had cardiac-related clinical signs present at the visit to the veterinary cardiology specialist. It remains unclear in this subgroup of dogs whether the appearance of clinical signs was the main reason for referral.

Aortic stenosis, pulmonic stenosis and left-to-right shunting patent ductus arteriosus were found to be the most common causes of a pathologic murmur in the present study, similarly to other reports on congenital cardiac anomalies [10]. In various published studies on left-to-right shunting patent ductus arteriosus, the median age of diagnosis and therapy was similar to our study, varying from two to 7.7 months (ranging between 1 month and 9 years and 7 months) [14, 24–32]. However, several studies on left-to-right shunting patent ductus arteriosus report a median age at diagnosis and therapy of around 1 year (ranging from 0.33 to 15 years) [16, 20, 33, 34], and one study reports a mean age of even 3.2 years (with a range of 2 months–12 years) [8]. Additionally, two papers describe findings on left-to-right shunting patent ductus arteriosus specifically in elderly dogs, whose median age at the diagnosis was 7.4 years (range 5.1–12.3) when dogs above 5 years of age were included, and a median age of 4 years (range 2–9 years) when dogs above 2 years of age were included [17, 21]. Regarding pulmonic stenosis, the other most common correctable congenital anomaly, a comparable conclusion can be drawn. Some studies report similar median age of diagnosis to our study, varying from 6 to 7.3 months of age (range 2–48 months) [30, 35–37]. However, several studies report a median age of about 1 year (range 2 months–16 years) [23, 38–41]. Similarly to the late diagnosis of patent ductus arteriosus, the median age when a pulmonic stenosis was diagnosed in some reports was surprisingly high, around 3 years (range 2 months–14 years) [8, 20, 42]. In our study, there was a median delay of 54 days between the diagnosis and the balloon valvuloplasty. This delay can be explained by the less urgent nature of the disease compared to the left-to-right shunting patent ductus arteriosus, and the time owners need to consider the costs, benefits and risks of the procedure, especially because balloon valvuloplasty is not a curative procedure as opposed to occlusion of a left-to-right shunting patent ductus arteriosus. Because of this delay of therapy after diagnosis, the referral must take place as early as possible after detecting a cardiac murmur, when the dogs are asymptomatic, young and when the large breed puppies are still as small as possible. The reported median age at diagnosis of less common and not routinely correctable congenital cardiac anomalies gives a similarly wide range as those of the correctable ones, such as 15 months (range 2–126) in case of tricuspid valve dysplasia [5] and 9 months (range 2.2 months–11.8 years) in case of ventricular septal defect [4]. Because subaortic stenosis is more a developmental than a pure congenital anomaly, diagnosing this condition might be delayed for this objective reason.

In the present study, the median age of a dog with a suspected congenital cardiac anomaly was 6.3 months old when it was examined by a veterinary cardiology specialist. The age when dogs with left-to-right shunting patent ductus arteriosus and severe pulmonic stenosis underwent a cardiac intervention in our study was among the lowest compared to published reports. However, if we take into consideration that based on our findings, puppies received their first vaccination and health check at a median age of 6.4 weeks, the delay in referral of more than three months to a veterinary cardiology specialist seems unnecessarily long. Because the second and third vaccinations routinely take place at 9 and 12 weeks of age together with repeated health examinations, the median age of 3.3 months seems still late for the first documentation of a murmur. This finding is especially interesting because the health examination at 9 and 12 weeks of age takes place by the new owner’s veterinarian, on an individual basis. The reason for the delay between the murmur documentation and the visit to the cardiology specialist remains unknown. Delay in referral can be caused by three parties: (1) by the referring veterinarian, who might not recommend immediate referral, (2) by the owner, who does not see the urgency of revealing the cause of a murmur, or (3) by the referring hospital. In one third of the referred dogs in the present study, the veterinary cardiology specialist recommended an interventional therapy for the congenital anomaly, which represents a relative high prevalence of severe and correctable anomalies. This finding emphasizes the importance of recommending referral to all owners of puppies with a murmur to a veterinary cardiology specialist, and preferably as soon as possible and ideally before the puppy is sold to the new owner.

How the echocardiographic findings of the non-cardiologist affected the decision about referral to a veterinary cardiology specialist remains speculative. Also, the number of dogs that after such an echocardiogram were not referred to a veterinary cardiology specialist remains unknown. Possible reasons why dogs with a suspected congenital cardiac anomaly were first referred to a non-cardiologist for echocardiography can have several reasons, such as lower examinations costs, shorter travel distance, and a possibly quicker and more flexible appointments. However, throughout the study period the cardiology service offered appointments with a veterinary cardiology specialist within a week of receiving the referrals.

The present study has several limitations. The first limitation is that the date when the murmur was documented for the first time was unknown for many dogs because of unavailable pet passports or medical records. This shortcoming results from the retrospective nature of the study. Another limitation is that in the study period three cardiologists performed the examinations, which might have affected the assessment of murmur intensity due to the subjective nature of this test. In addition, comparing murmur intensity of two veterinarians based on retrospectively gained recorded findings is less reliable because the auscultations took place on different days (sometimes with a long time interval in between) and under different circumstances, moreover it can be effected by the natural history of the disease. Finally, the major question why puppies with a cardiac murmur are not immediately referred to a veterinary cardiology specialist remained unanswered.

## Conclusion

Only 10% of the puppies in the study were referred to a veterinary cardiology specialist for murmur investigation before they were sold to a new owner. Referral prior to re-homing would have been feasible if the murmur had been detected and documented by the breeder’s veterinarian, if referral was offered by the breeder’s veterinarian and the referral accepted by the breeder. This study emphasizes the importance of early referral, to reduce the medical, financial and emotional consequences of delayed murmur investigation for all parties involved. The reasons for delayed referral to veterinary cardiology specialists remain undetermined. However, the importance of early referral is clear, as the vast majority of the population had congenital defects, and as interventional procedures to correct severe defects were recommended for about a third of the dogs in this study. When murmur investigation is carried out prior to re-homing, new owners can make an informed decision when taking on a puppy with congenital heart disease.

## Data Availability

The datasets used and/or analysed during the current study are available from the corresponding author on reasonable request.
